# Gender Difference in the Epidemiological Association between Metabolic Syndrome and Olfactory Dysfunction: The Korea National Health and Nutrition Examination Survey

**DOI:** 10.1371/journal.pone.0148813

**Published:** 2016-02-09

**Authors:** Se-Hwan Hwang, Jun-Myung Kang, Jae-Hyun Seo, Kyung-do Han, Young-Hoon Joo

**Affiliations:** 1 Department of Otolaryngology-Head and Neck Surgery, The Catholic University of Korea, Seoul, Korea; 2 Department of Biostatistics, College of Medicine, The Catholic University of Korea, Seoul, Korea; Indiana University Richard M. Fairbanks School of Public Health, UNITED STATES

## Abstract

Metabolic syndrome (MetS) is associated with a higher risk of morbidity and/or mortality for various chronic diseases. The aim of this study was to investigate the relationships of MetS and its components with olfactory dysfunction in a representative Korean population. We analyzed the data from the Korean National Health and Nutrition Examination Survey (2008–2010). A total of 11,609 adults who underwent otolaryngological examination were evaluated. The olfactory function was classified as normosmia or hyposmia by a self-report questionnaire according to the sense problems of smell during the past 3 months. MetS was diagnosed if a participant had at least three of the following: (1) WC ≥90 cm in men and ≥80 cm in women; (2) fasting blood sugar ≥ 100 mg/dL or medication use for elevated glucose; (3) fasting triglyceride ≥ 150 mg/dL or cholesterol-lowering medication use; (4) HDL-cholesterol <40 mg/dL in men and <50 mg/dL in women or cholesterol-lowering medication use; and (5) SBP ≥ 130 mmHg and/or DBP ≥ 85 mmHg or antihypertensive drug use for patients with a history of hypertension. The prevalence of olfactory dysfunction in the study population was 6.3%. The prevalence of olfactory dysfunction was significantly higher in older people with MetS than in those without MetS in both sexes (male, 42.0 ± 3.4% vs. 34.7 ± 0.9%, p = 0.0354; female, 46.2 ± 2.8% vs. 37.8 ± 0.8%, p = 0.0026). However, elevated waist circumference, elevated fasting glucose, elevated triglycerides, reduced HDL cholesterol, elevated blood pressure, severe stress, depressed mood, and suicidal ideation were significantly associated with olfactory dysfunction only in women. After controlling for confounders, olfactory dysfunction was significantly associated with MetS (odds ratio, 1.352; 95% confidence interval, 1.005–1.820) only in women. MetS are associated with olfactory dysfunction only in Korean women.

## Introduction

Metabolic syndrome (MetS) is a condition of unknown cause that presents with symptoms of insulin resistance, obesity, hypertension, dyslipidemia, and systemic inflammation; it represents a healthcare epidemic in Western industrialized countries [1]. In 2009, the US Department of Health and Human Services estimated the prevalence of MetS to be 35.1% in men and 32.6% in women aged 20 years or older. Likewise, in 2012, the Korea National Health Insurance Corporation reported the prevalence of MetS in Korean men and women aged over 30 years was 31.4% and 18.4%, respectively, which continues to increase annually. These findings indicate MetS is becoming a serious public health issue in both the US and Korea [2].

It was recently proposed that the olfactory system may serve a secondary function as an internal sensor of chemical state or nutritional balance. During the past decade, the peripheral and central neuronal pathways in the olfaction functions has been known to be modulated responding to the circulating levels of various molecules, including leptin, insulin, and glucose, which are associated with nutritional status insulin, and glucose.[3] MetS components such as high glucose, high triglyceride, and high blood pressure have been reported to be associated with olfactory dysfunction, separately. However, there has been no study to evaluate the direct association between MetS and olfactory dysfunction.[3–8] Considering the above mentioned, it would be worthwhile to determine whether there is a relationship between MetS and olfactory dysfunction as well as a correlation between lower olfactory scores and the presence of MetS components such as large waist circumference (WC), high glucose, high triglyceride, low HDL cholesterol, and high blood pressure (BP). The 2010 Korean National Health and Nutrition Examination Survey (KNHANES), a government-driven survey of the Korea Center for Disease Control and Prevention, was the first population-based study to assess olfactory dysfunction as well as MetS components in the non-institutionalized civilian population of South Korea [9]. Accordingly, the present determined the prevalence of olfactory dysfunction and its associations with MetS component by using the KNHANES data.

## Materials and Methods

### Ethics Statement

The study protocol was approved by the Institutional Review Board of the Catholic Medical Center.

### Study Population

This study was based on data collected during the 2008–2010 KNHANES. The KNHANES is a nationwide survey conducted by the Division of Chronic Disease Surveillance under the Korea Centers for Disease Control and Prevention since 1998 that is designed to accurately assess national health and nutrition levels. A field survey team that included an otolaryngologist and nurse examiners for health assessments moved with a mobile examination unit and performed interviews and physical examinations. The survey consists of a health interview, nutritional survey, and health examination survey. The survey amasses data through household interviews and direct standardized physical examinations conducted in specially equipped mobile examination centers. The KNHANES methodology has been described in detail elsewhere [10–12].

The sample included 11,609 participants aged over 19 years. We focused on this population because age-related olfactory dysfunction becomes progressively worse in adults ≥ 19 years. Written informed consent was obtained from all participants prior to the survey.

### Survey for olfactory dysfunction

Participants aged ≥19 years were examined. The olfactory questionnaire inquired about whether the participants have had problems with their sense of smell during the past three months (S1 Table). Participants with positive and negative responses were considered hyposmic and normosmic, respectively.

### Lifestyle habits

Data on medical history and lifestyle habits were collected using self-reported questionnaires. Patients were categorized according to smoking history as current smokers, ex-smokers, or nonsmokers. Participants who drank more than 30 g alcohol/day were designated heavy drinkers. Regular exercise was defined as strenuous physical activity performed for at least 20 min at a time at least three times per week. Job was defined as the employed state and participants who had life partners spouse were designated as spouse. Physical and mental health status included levels of perceived stress (‘‘light or no” or ‘‘some or heavy”), experience of feeling depressed for at least 2 weeks (yes, no), suicidal ideation for the last 12 months (yes, no), and self-rated health status (excellent or good, fair, and poor or very poor).

### Anthropometric and laboratory measurements

Weight and height were measured by well-trained medical professionals. Standing height was measured when the participant faced directly ahead with shoes taken off; feet together; arms by their sides; and heels, buttocks, and upper back in contact with the wall. Height was measured in centimeters to one decimal point using a SECA 225 (SECA, Germany). WC was measured to the nearest 0.1 cm at the level of the midpoint between the iliac crest and costal margin at the end of a normal expiration. Weight was measured using a GL-6000-20 scale (Cass, Korea) in kilograms to one decimal point. BMI was calculated as weight (kg) / height (m^2^). Obesity was defined as BMI ≥ 25 kg/m^2^. International Obesity Task Force (IOTF) and the World Health Organization (WHO) Regional Office for the Western Pacific Region recommend defining obesity in Asians as those with a BMI ≥25 kg/m^2^. [13] BP was measured while the participant was in a sitting position following a 5-minute rest period. Systolic blood pressure (SBP) and diastolic blood pressure (DBP) were measured by a mercury sphygmomanometer (Baumanometer, W. A. Baum Co., Copiague, NY, USA) on the right arm. To assess serum levels of biochemical markers, blood samples were obtained from the antecubital veins of the participants following a 10–12-hour overnight fast. Serum levels of fasting blood sugar, total cholesterol, triglyceride, high-density lipoprotein (HDL) cholesterol, and low-density lipoprotein (LDL) cholesterol were measured using an enzymatic method (Hitachi Automatic Analyzer 7600, Hitachi, Tokyo, Japan).

### MetS definition

MetS was defined according to the criteria proposed by the American Heart Association and the National Heart, Lung, and Blood Institute together with the International Diabetes Federation in 2009.[14] MetS was diagnosed if a participant had at least three of the following: (1) WC ≥90 cm in men and ≥80 cm in women according to the International Diabetes Federation criteria for Asian countries; (2) fasting blood sugar ≥ 100 mg/dL or medication use for elevated glucose; (3) fasting triglyceride ≥ 150 mg/dL or cholesterol-lowering medication use; (4) HDL-cholesterol <40 mg/dL in men and <50 mg/dL in women or cholesterol-lowering medication use; and (5) SBP ≥ 130 mmHg and/or DBP ≥ 85 mmHg or antihypertensive drug use for patients with a history of hypertension.

### Statistical analysis

Statistical analyses were performed using the SAS survey procedure (version 9.3; SAS Institute, Cary, NC, USA) to reflect the complex sampling design and sampling weights of the KNHANES and provide nationally representative prevalence estimates. The procedures included unequal probabilities of selection, oversampling, and nonresponse so that inferences could be made about the Korean adolescent participants.

The prevalence and 95% confidence intervals (CIs) for olfactory dysfunction were calculated. For univariate analysis, the Rao–Scott χ^2^ test (using PROC SURVEYFREQ in SAS) and logistic regression analysis (using PROC SURVEYLOGISTIC in SAS) were used to determine the associations between olfactory dysfunction and risk factors in a complex sampling design. Continuous and categorical variables were described using means and standard errors, and numbers and percentages, respectively. Logarithmic transformation was used to analyze variables with skewed distributions. Multivariate logistic regression analyses were used to determine the association between olfactory dysfunction and MetS. We first adjusted for age (model 1) and then adjusted for the variables in model 1 plus smoking status, alcohol intake, regular exercise, house income, and education level (model 2), and then for model 2 plus stress, and depressed mood (model 3). P-values were two-tailed, and the level of significance was set at P < 0.05.

## Results

### General characteristics of the study population

Among the 11,609 participants ≥19 years old, 780 had experienced olfactory dysfunction; thus, the prevalence of olfactory dysfunction was 6.3% (men, 5.8% and women, 6.7%). The mean age of those with olfactory dysfunction was significantly higher than those without olfactory dysfunction (59.8±0.5 vs. 55.1±0.2, p < 0.0001). The baseline characteristics of the study participants according to olfactory dysfunction are shown in Table 1. Both men and women with olfactory dysfunction were significantly more likely to be older. Job, education level, house income, DBP, total cholesterol, LDL cholesterol, fat intake, and poor self-rated health were significantly associated with olfactory dysfunction in men. Meanwhile, spouse, education level, house income, WC, SBP, HDL cholesterol, triglyceride, fat intake, severe stress, depressed mood, suicidal ideation, and poor self-rated health were significantly associated with olfactory dysfunction in women.

**Table 1:** Analysis of factors potentially associated with olfactory dysfunction (n = 11609).

Parameter	Male			Female		
	Yes (n = 327)	No (n = 4727)	P-value	Yes (n = 453)	No (n = 6102)	P-value
**Age (years)**	58.0±0.8	54.4±0.2	<0.0001*	61.2±0.7	55.8±0.2	<0.0001*
**Current Smoker (%)**	36.7±3.6	38.5±0.9	0.6385	5.4±1.5	4.1±0.3	0.3661
**Heavy drinker (%)**	15.2±2.7	18.1±0.7	0.3253	1.3±0.8	1.1±0.2	0.7165
**Routine exercise (%)**	19.3±3.1	22.2±0.8	0.3821	15.9±2.2	17.2±0.7	0.5997
**Job (%)**	71.2±3.0	81.5±0.7	<0.0001*	50.1±3.0	52.0±0.9	0.5296
**Spouse (%)**	92.0±2.4	92.2±0.5	0.9134	70.4±2.8	77.2±0.7	0.0093*
**Residential area; Rural (%)**	30.9±4.6	24.6±2.0	0.0901	33.0±3.9	23.1±1.9	0.0009
**Education; ≥ High school (%)**	52.3±3.5	65.2±1.1	0.0002*	30.5±2.7	46.4±1.0	<0.0001*
**Income; lower quartile (%)**	23.3±2.8	16.6±0.7	0.0091*	31.5±2.8	22.5±0.8	0.0005*
**Waist circumference (cm)**	85.2±0.6	85.2±0.2	0.9729	82.5±0.5	80.6±0.2	0.0002*
**Body mass index (kg/m**^**2**^**)**	23.8±0.2	24.1±0.1	0.2076	24.4±0.2	24.0±0.1	0.0793
**Systolic BP (mmHg)**	122.9±1.1	124.1±0.3	0.2814	124.5±1.0	121.8±0.3	0.0130*
**Diastolic BP (mmHg)**	77.9±0.7	80.8±0.2	<0.0001*	75.6±0.6	76.0±0.2	0.4295
**Glucose (mM)**	102.9±1.6	103.3±0.5	0.7898	99.4±1.2	98.6±0.4	0.5258
**Total cholesterol (mM)**	181.5±2.4	191.1±0.7	0.0002*	197.1±1.8	197.3±0.6	0.8989
**HDL cholesterol (mM)**	47.0±0.7	48.9±0.2	0.0123	51.6±0.6	53.8±0.2	0.0008*
**LDL cholesterol (mM)**	103.5±2.3	111.0±0.6	0.0024*	119.6±1.9	118.6±0.5	0.6244
**Triglyceride (mM)**	133.4(123.0–144.6)	136.9 (133.8–140.1)	0.547	116.3(109.9–123.1)	107.1(105.2–109.1)	0.0071*
**Energy/1 day (kcal/d)**	2271.2±63.7	2357.7±20.1	0.209	1608.6±41.2	1666.2±12.3	0.197
**Fat intake/1 day (g/d)**	15.6±0.7	17.1±0.2	0.0480*	12.4±0.4	15.1±0.1	<0.0001*
**Severe stress (%)**	6.3±0.9	5.6±0.5	0.434	8.3±0.9	6.0±0.5	0.0120*
**Depressed mood (%)**	6.7±1.4	5.6±0.5	0.46	9.2±1.1	6.0±0.5	0.0021*
**Suicidal ideation (%)**	7.3±1.3	5.5±0.5	0.133	9.3±1.1	6.0±0.4	<0.0001*
**Poor self-rated health (%)**	9.5±1.3	5.0±0.5	<0.0001*	10.2±0.9	5.5±0.5	<0.0001*

Values are mean ± SE or % ± SE.

Triglyceride levels are presented as geometric mean (95% confidence interval).

* Significant at p<0.05

### Prevalence of olfactory dysfunction with respect to MetS and its components

Table 2 shows the prevalence of MetS and its components according to the olfactory dysfunction by gender. The prevalence of MetS was significantly higher in participants with olfactory dysfunction than that in those without olfactory dysfunction for both sexes (men, 42.0 ± 3.4% vs. 34.7 ± 0.9%, p = 0.0354 and women, 46.2 ± 2.8% vs. 37.8 ± 0.8%, p = 0.0026). The prevalence of all MetS components was higher in the presence of olfactory dysfunction in women but was similar in the presence of olfactory dysfunction in men.

**Table 2:** Prevalence of metabolic syndrome and its components according to the presence or absence of olfactory dysfunction by gender.

Parameter	Male			Female		
	Presence	Absence	P-value	Presence	Absence	P-value
**Presence of metabolic syndrome (%)**	42.0±3.4	34.7±0.9	0.0354*	46.2±2.8	37.8±0.8	0.0026*
**High waist circumference (%)**	32.6±3.1	27.5±0.9	0.1043	58.4±2.6	50.5±0.9	0.0034*
**High glucose (%)**	46.0±3.6	41.6±0.9	0.2143	36.3±2.8	30.3±0.8	0.0277*
**High triglyceride (%)**	42.6±3.2	45.8±0.9	0.3368	40.4±2.9	33.0±0.8	0.0106*
**Low HDL cholesterol (%)**	31.6±3.3	26.0±0.8	0.0865	50.6±2.7	44.9±0.8	0.0443*
**High blood pressure (%)**	53.8±3.5	54.7±0.9	0.1043	54.3±3.0	45.6±0.9	0.0056*

Values are % ± SE.

* Significant at p<0.05

Metabolic syndrome was diagnosed if a participant had at least three of the following; (1) high waist circumference, (2) High glucose, (3) High triglyceride, (4) Low HDL cholesterol, and (5) High blood pressure.

High waist circumference, waist circumference ≥90 cm (men) or 85 cm (women); High glucose, fasting blood sugar ≥ 100 mg/dL or medication use for elevated glucose; High triglyceride, fasting triglyceride ≥ 150 mg/dL or cholesterol-lowering medication use; Low HDL cholesterol, HDL-cholesterol <40 mg/dL (men) or <50 mg/dL (women) or cholesterol-lowering medication use; High blood pressure, systolic blood pressure ≥ 130 mmHg and/or diastolic blood pressure ≥ 85 mmHg or antihypertensive drug use.

### Associations between olfactory dysfunction and MetS

Table 3 shows the degree of association with the prevalence of olfactory dysfunction and MetS component in both sexes after adjusting for confounders. The adjusted odds ratio (OR) for olfactory dysfunction was not significant in men with MetS. Meanwhile, the risk of olfactory dysfunction was significantly associated with MetS (OR [95% CI]: 1.352 [1.005–1.820] in model 2 and 3) in only women after adjusting for confounders.

**Table 3:** Logistic regression models of metabolic syndrome for olfactory dysfunction by gender.

Parameter	Odds ratio (95% Confidence interval)					
	Male			Female		
	Model 1	Model 2	Model 3	Model 1	Model 2	Model 3
**Presence of metabolic syndrome**	1.042 (0.808–1.343)	1.031 (0.796–1.334)	1.030 (0.795–1.334)	1.319 (0.990–1.758)	1.352 (1.005–1.820)	1.352 (1.005–1.820)
**High waist circumference**	1.163 (0.928–1.457)	1.190 (0.928–1.457)	1.200 (0.934–1.541)	1.259 (0.942–1.682)	1.305 (0.970–1.757)	1.299 (0.965–1.748)
**High glucose**	1.064 (0.823–1.375)	0.913 (0.678–1.230)	0.970 (0.672–1.225)	1.119 (0.846–1.480)	0.892 (0.675–1.179)	0.891 (0.674–1.177)
**High triglyceride**	1.150 (0.886–1.492)	1.137 (0.876–1.476)	1.134 (0.873–1.473)	0.936 (0.713–1.229)	0.960 (0.725–1.271)	0.960 (0.724–1.272)
**Low HDL cholesterol**	1.046 (0.831–1.318)	1.084 (0.855–1.374)	1.078 (0.850–1.367)	1.256 (0.916–1.722)	1.233 (0.892–1.704)	1.235 (0.894–1.706)
**High blood pressure**	0.946 (0.712–1.257)	1.063 (0.808–1.397)	1.055 (0.800–1.391)	0.852 (0.646–1.123)	1.095 (0.821–1.459)	1.096 (0.822–1.460)

Model 1: Adjusted for age.

Model 2: Adjusted for age, smoking status, alcohol intake, regular exercise, house income, education level.

Model 3: Adjusted for age, smoking status, alcohol intake, regular exercise, house income, education level, stress, depressed mood.

## Discussion

Although metabolic risk factors including abdominal obesity, hyperglycemia, and dyslipidemia are closely associated with olfactory function, no study has evaluated the relationship between MetS and olfactory dysfunction in the general population. In the present study looking at the association of MetS with olfactory dysfunction in the Korean people, we found that MetS (OR,1.352 in model 2 and 3) was significantly associated with olfactory dysfunction in only women, after adjusting for risk factors including mental health such as stress and depressed mood. However, despite the similar tendency of association with all MetS components and olfactory dysfunction in the both sex, there were statistical significance of the association between the prevalence of olfactory dysfunction and MetS components in only the women.

Obesity used to be related to genetic conditioning and linked, first of all, to high energy nutrition. Recently, there have been the studies to evaluate the causal link between olfactory sensing and obesity. Hubert et al and Obrebowski et al found that obesity increased the odor detection threshold and decreases the ability to discriminate and identify odors in both adults and children [6, 15]. Previous studies have implemented various measurements including BMI, hip-to-waist ratio, and WC to investigate the impacts of obesity on the brain and cognitive health. Among these measurements, WC is strongly associated with prolonged P3 latencies of olfactory event-related potentials (i.e., slower cognitive processing), which is an objective measure of the sensory-perceptual processing of odor stimuli [16]. These results may be concordant with the present result that people with olfactory dysfunction had larger WC than those without olfactory dysfunction.

High-fat diet, regardless of the severity of obesity, could affect the general neuro-architecture of the olfactory system [3]. Consuming a high-fat diet starting at a young age leads to hyperlipidemia, obesity development, and progression to MetS [17]. A high-fat diet-induced mouse model of obesity shows a clear reduction in olfactory discrimination wherein obese mice have at least a 20% reduction in the number of correct decisions compared to mice fed a control-fat diet. Furthermore, chronic exposure to dietary fat reduces action potential firing frequency and inter-spike intervals of mitral cells of the olfactory bulb, EOG amplitude, and the abundance of olfactory sensory neurons and their axonal projections [3, 7]. In humans, high-fat diets and obesity have been shown to impair learning and memory, which are required for odor threshold discrimination (i.e., two-choice paradigm) [7]. Therefore, the present results regarding the prevalence of olfactory dysfunction with respect to dyslipidemia are of particular relevance to hyperlipidemia-related functional changes of olfaction and cognition.

The olfactory system was recently reported to be connected with the endocrine system, and there is increasing evidence of the roles of insulin, ghrelin, and leptin in the modulation of olfaction [4]. In particular, previous studies report a relationship between diabetes mellitus (DM) and olfactory dysfunction as well as a correlation between lower olfactory scores and the presence of diabetic complications.[4] Many patients with DM have impaired olfaction, the physiological basis of which is unknown. As mentioned above, several studies demonstrate a relationship between DM and olfactory dysfunction as well as a correlation between lower olfactory scores and the presence of diabetic complications. Some authors even suggest olfactory tests could be used for the early recognition of diabetic complications [18]. The olfactory area in rats is particularly rich in insulin receptors and insulin receptor mRNA; accordingly, abnormalities of insulin receptor function in the olfactory area may manifest as olfactory disturbances [8]. Both macrovascular and microvascular diseases complicated by DM are proposed to be underlying mechanisms explaining olfactory dysfunction in DM.[4] In the present study, olfactory dysfunction was more frequent in participants with hyperglycemia than in participants without hyperglycemia. This finding suggests that the blood glucose level should also be considered when evaluating the olfactory system.

Patients with a loss of smell are reported to use larger quantities of salt, thereby increasing the risk of hypertension. Odors are reported to increase salt intensity by as much as 25%, suggesting that a loss of odor perception may be associated with decreased perception of salt intensity. In addition, hypertensive patients prefer saltier fluids than controls and are less sensitive to the taste of salt, consequently consuming more of it [5]. Considering the increasing salt uptake of people with olfactory dysfunction and the association between salt intake and hypertension, the association between hypertension and olfactory dysfunction in the present study is consistent with the literature. Although metabolic risk factors were significantly associated with olfactory dysfunction in the present study, no study has examined how MetS is affected by olfactory dysfunction.

The reason for gender difference between MetS and olfactory dysfunction is not clear but a few studies have reported associations of some components of MetS with olfactory dysfunction among women. This gender specific association also might be explained by differences of hormonal characteristics of MetS between men and women [19]. And low levels of physical activity are strongly associated with the development of MetS and chronic diseases [20]. There are also sex differences in stress sensitivity and stress-related behaviors. For example, women have increased stress sensitivity, exhibiting a higher-magnitude hormonal response and delayed stress recovery time compared to men. In addition, sex differences in motivation for high-calorie foods have been observed in both normal-weight and obese individuals; specifically, women prefer high-fat foods more frequently than men [21]. These results potentially suggest that a possible sex-specific influence of stress on disease vulnerability may result in differences in the strength of the relationship between olfactory dysfunction and MetS components.

Previously, Lee et al used the data from the 2009 KNHANES and demonstrated that several medical conditions such as hypertension, low HDL, and cholesterolemia were significantly associated with olfactory dysfunction in Korean population. However, the level of triglyceride and glucose were not associated with olfactory dysfunction. The waist circumference and the difference of association according to the gender factor were not measured in their study [22]. These results were somewhat different from our results that the prevalence of olfactory dysfunction in only female was significantly higher in the presence of all MetS components including high waist circumference, high glucose, high triglyceride, low HDL cholesterol, and high blood pressure. In our study, we measured the prevalence of olfactory dysfunction and its associations with MetS component by using the 2010 KNHANES data. The discrepancy between our study and their study would be due to the enrolled data and the evaluation according to the gender factor.

To our knowledge, the present study is the first large population-based study showing that MetS is associated with olfactory dysfunction. However, the present study has some limitations that should be mentioned. First, there may be some response biases on the reporting of several parameters such as mental status, stress level, and olfactory dysfunction, because the KNHANES was conducted using self-administered questionnaires. However, as otorhinolaryngological questionnaires were administered by trained otorhinolaryngology residents, the determination of olfactory dysfunction may be clinically quite relevant [22]. And in this cohort the mean age was higher in women than in men. Age differences in the sexes might contribute to the associations between MetS and olfactory dysfunction. Second, as the present study was cross-sectional, the causal relationship of the risk factors with olfactory dysfunction remains inconclusive. Nevertheless, the results may be reliable, because this was a nationwide population-based study [9]. Third, the related variables of which the presence or absence may be associated with olfactory dysfunction and could affect the results were not able to be evaluated and the participants with other acute disease status, such as flu and rhinitis, were not able to be excluded from this study. However, KNHANES is a nationwide cross-sectional study to select a representative sample of the Korean population. This survey employed stratified multi-stage design based on age, sex, and residence geographic area. The characteristics such as the nationwide population-based study would overcome the limitation related the acute disease status [22, 23]. Additionally, we evaluated the direct association between MetS and olfactory dysfunction as well as the unknown separate relationship between components of MetS such as hypertension and olfactory dysfunction. We also performed the evaluation of the change of these relationships according to the gender factor. Because this study was based on the KNHANES, this characteristic could make our study perform more unbiased assessment of the relationship about olfactory disorders in the general population compared with previous studies which evaluated the olfactory disorders in clinical populations or those seeking care (i.e., patients submitting for nasal surgery, etc.).

Despite these shortcomings, the present study is the first population-based study to assess the relationship between olfactory dysfunction and MetS in the general Korean population. Our results could suggest that clinician need to consider the olfactory function of those with MetS or metabolic risk factors. In future, further longitudinal studies or well controlled studies would be helpful to understand the complex relationship between olfactory dysfunction and MetS.

## Conclusion

Data from the 2010 KNHANES indicate MetS are associated with olfactory dysfunction in women. The results of this general population-based study on the prevalence of olfactory dysfunction and its relationship with MetS could contribute to their prevention and management in the Korean population.

## Supporting Information

S1 Table Korea National Health and Nutrition Examination Survey Questionnaire item assessing olfactory disorders. (DOC)
