# Supplementary material for: Gender Difference in the Epidemiological Association between Metabolic Syndrome and Olfactory Dysfunction: The Korea National Health and Nutrition Examination Survey
Source: PLoS One. 2016 Feb 9;11(2):e0148813. doi: 10.1371/journal.pone.0148813 (PMC4747555; doi:10.1371/journal.pone.0148813)
Supplement: S1 Table — (DOC) [file pone.0148813.s001.doc]

# Table S1. Korea National Health and Nutrition Examination Survey Questionnaire item assessing olfactory disorders

| Had problems with symptoms related smell more than 3 months during past 12 months? | | |
| --- | --- | --- |
| Symptom | Yes | No |
| Anterior/posterior nasal drip  Nasal obstruction  Facial pain or pressure  Anosmia or hyposmia |  |  |

Values are mean ± SE

* Significant at p<0.05
